# A New Image Encryption Algorithm Based on Chaos and Secure Hash SHA-256

**DOI:** 10.3390/e20090716

**Published:** 2018-09-19

**Authors:** Shuqin Zhu, Congxu Zhu, Wenhong Wang

**Affiliations:** 1 School of Computer and Science, Liaocheng University, Liaocheng 252059, China; 2 School of Information Science and Engineering, Central South University, Changsha 410083, China; 3 School of Physics and Electronics, Central South University, Changsha 410083, China; 4 Guangxi Colleges and Universities Key Laboratory of Complex System Optimization and Big Data Processing, Yulin Normal University, Yulin 537000, China

**Keywords:** chaotic system, image encryption, permutation-diffusion, SHA-256 hash value, dynamic index

## Abstract

In order to overcome the difficulty of key management in “one time pad” encryption schemes and also resist the attack of chosen plaintext, a new image encryption algorithm based on chaos and SHA-256 is proposed in this paper. The architecture of confusion and diffusion is adopted. Firstly, the surrounding of a plaintext image is surrounded by a sequence generated from the SHA-256 hash value of the plaintext to ensure that each encrypted result is different. Secondly, the image is scrambled according to the random sequence obtained by adding the disturbance term associated with the plaintext to the chaotic sequence. Third, the cyphertext (plaintext) feedback mechanism of the dynamic index in the diffusion stage is adopted, that is, the location index of the cyphertext (plaintext) used for feedback is dynamic. The above measures can ensure that the algorithm can resist chosen plaintext attacks and can overcome the difficulty of key management in “one time pad” encryption scheme. Also, experimental results such as key space analysis, key sensitivity analysis, differential analysis, histograms, information entropy, and correlation coefficients show that the image encryption algorithm is safe and reliable, and has high application potential.

## 1. Introduction

In recent years, with the rapid development of computer technology, digital image processing technology has also rapidly developed and penetrated into all aspects of life, such as remote sensing, industrial detection, medicine, meteorology, communication, investigation, intelligent robots, etc. Therefore, image information has attracted widespread attention. Image data security is very important, especially in the special military, commercial and medical fields. Image encryption has become one of the ways to protect digital image transmission. However, the image data has the characteristics of large amounts of data, strong correlation and high redundancy, which lead to low encryption efficiency and low security, so the traditional encryption algorithms, such as Data Encryption Standard (DES) and Advanced Encryption Standard (AES), cannot meet the needs of image encryption [1]. Chaos has the characteristics of high sensitivity to the initial conditions and system parameters, no periodicity, pseudo randomness, ergodicity and chaotic sequences can be generated and regenerated accurately, so it is especially suitable for image encryption. Therefore, many image encryption algorithms have been put forward using chaotic system. In 1998, the American scholar Fridrich put forward the classical substitution-diffusion architecture for image encryption [2]. This structure subsequently has drawn world-wide concern, and nowadays, most of the image encryption schemes based on chaos adopt this structure and achieved satisfactory encryption effect, such as pixel-level scrambling approaches [3,4,5], enhanced diffusion schemes [6], improved hyper-chaotic sequences [7], linear hyperbolic chaotic system [8], and bit-level confusion methods [9,10,11]. However, only using low dimensional chaotic system to encrypt images cannot guarantee enough security. Some works on cryptanalysis [12,13,14,15,16,17,18] show that many chaos-based encryption schemes were insecure, and the main reason is that the encryption key has nothing to do with the plaintext. For examples, an image encryption algorithm with only one round diffusion operation is proposed in [19]. The algorithm has the advantages of easy implementation, low complexity and high sensitivity to cyphertext and plaintext, but Diab et al. [20] cryptanalyzed this algorithm and broke the algorithm with only one chosen plaintext. Akhavan et al. [21] cryptanalyzed an image encryption algorithm based on DNA encoding and the curve cryptography and found that the algorithm cannot resist chosen plaintext attacks. Using a skew tent chaotic map, Zhang [22] proposed a novel image encryption method, which adopted a cyphertext feedback mechanism to resist chosen plaintext attacks, but Zhu et al. [23] cracked the algorithm by applying a chosen plaintext combined with chosen cyphertext attack. Various plaintext-related key stream generation mechanisms have been proposed to improve the ability to resist chosen plaintext attacks [24,25,26,27]. In most of these algorithms, the SHA-256 hash value of image is used as the external key of the encryption system, so that the encryption keys of different images are different, so as to achieve the effect of “one time pad”. Taking the scheme in [28] as an example, firstly, the initial values and parameters of the two-dimensional Logistic chaotic map are calculated from the SHA 256 hash of the original image and given values. Secondly, the initial values and system parameters of the chaotic system are updated by using the Hamming distance of the original image. So the generated random sequence is related to the plaintext image. This encryption method has the advantages of high sensitivity to plaintext and strong attack against plaintext. However, the decryption end needs not only the initial key which is not related to the plaintext, but also the key related to the plaintext. Therefore, decrypting different cyphertext requires different plaintext-related keys, which essentially makes the system work in OTP fashion and greatly increases the complexity for applications.

Concerned about the above issue, we propose to encrypt images based on permutation–diffusion framework using secure hash algorithm SHA-256. Two innovations are the main contributs of this work. Firstly, the hash value of the plaintext image is converted into the number in the range of [0, 255], which is added as the random number around the plaintext image, rather than as the external key of encryption system. This can resist chosen plaintext attacks, and does not need the hash value of the plaintext image in the decryption phase. Secondly, in the permutation and diffusion processes, the generation of random sequences is related to intermediate cyphertext. In this way, the key used to encrypt different images is the initial value of the chaotic system, but the generated key stream is different.

## 2. Preliminaries

### 2.1. Adding Surrounding Pixels

A hash function is any function that can be used to map data of arbitrary size to data of a fixed size. Here, we use SHA-256 to generate the 256-bit hash value *V*, which can be divided into 32 blocks with the same size of 8-bit, the *i*-th block *v_i_*∈ [0, 255], *i* = 1, 2, …, 32, so *V* can be expressed as *V* = *v*_1_, *v*_2_, …, *v*_32_. Suppose the size of the plain-image *P* is *m* × *n*, obtain an integer *k* as:

(1) k=fix(2(m+n+1)/32)+1

where, *fix*(*x*) rounds the elements of *x* to the nearest integers towards zero. Then we generate a sequence *H* that has (32*k*) elements by:

(2) H=repmat(V, [1, k])

where, *repmat*(*V*, [1, *k*]) creates a large matrix *H* consisting of a 1 × *k* tiling of copies of *V*, e.g., *repmat*([3, 6, 9], [1, 2]) = [3, 6, 9, 3, 6, 9]. Then, matrix *RI* of size 2 × (*n* + 2) is formed by taking the first 2*n* + 4 numbers of the sequence *H*, and the *CI* matrix of size 2 × *m* is formed by taking the remaining 2*m* numbers of *H*. The elements of *RI* and *CI* have the same representation format as the pixels of *P*. For example, The SHA-256 hash value of the plaintext image “cameraman” of size 256 × 256 is the character string S, which is: S = “d6f35e24b1f70a68a37c9b8bfdcd91dc3977d7a98e67d453eb6f8003b6c6 9443”.

According to the string S, we can get a sequence *V* of length 32. *V* = (214, 243, 94, 36, 177, 247, 10, 104, 163, 124, 155, 139, 253, 205, 145, 220, 57, 119, 215, 169, 142, 103, 212, 83, 235, 111, 128, 3, 182, 198, 148, 67). So, the sequence *H* of length 1028 can be obtained as *H* = (214, 243, 94, 36, 177, 247, 10, …, 214, 243). Similarly, matrices *RI* and *CI* are also obtained, as shown below:

$$RI={\left(\begin{array}{l} \begin{array}{cccc} 214 & 243 & 94 & \ldots \begin{array}{ccc} \;148 & 67 & 214 \end{array} \end{array}\\ \begin{array}{cccc} 243 & 94\; & 36 & \ldots \begin{array}{ccc} \;67 & 214 & 243 \end{array} \end{array} \end{array}\right)}_{2\times (256+1)}\;CI={\left(\begin{array}{l} \begin{array}{cccc} 94 & 36 & 177 & \ldots \;\begin{array}{ccc} 67 & 214 & 243 \end{array} \end{array}\\ \begin{array}{cccc} 94 & 36 & 177 & \ldots \;\begin{array}{ccc} \;67 & 214 & 243 \end{array} \end{array} \end{array}\right)}_{2\times (256+1)}$$

**Figure 1:**
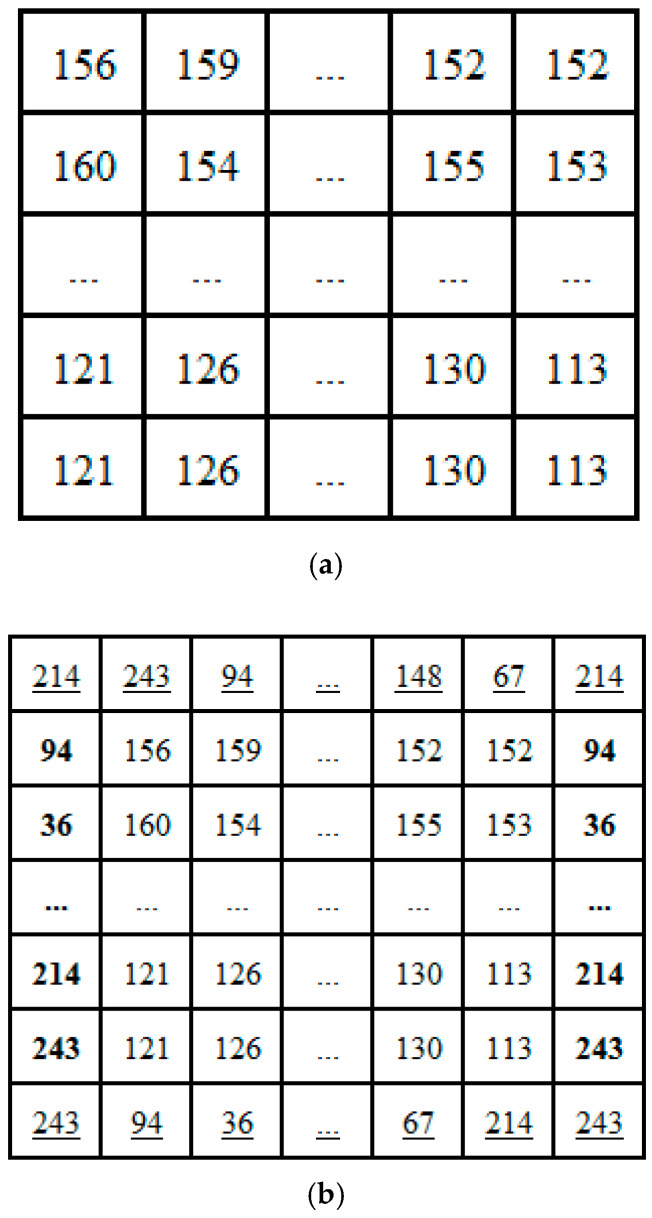
An example of adding surrounding pixels. (**a**) plain-image *P*; (**b**) operation result.

*RI* and *CI* will surround the plaintext image. These values will affect all pixels after the confusion and diffusion operation. Figure 1 shows a numerical example of using *RI* and *CI* to add pixels to the image “cameraman”. Figure 1b shows the result of the operation. It can be seen that the underscore is derived from *RI* and the value of bold is from *CI*.

### 2.2. Hyper-Chaotic System and Chebyshev Map

**Figure 2:**
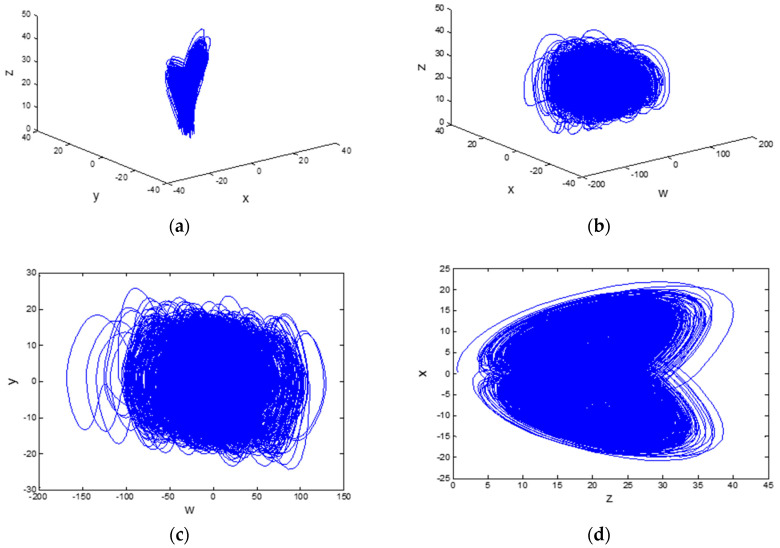
Hyper-chaotic attractor. (**a**) (x-y-z) plane; (**b**) (w-x-z) plane; (**c**) (w-y) plane; (**d**) (x-z) plane.

The scheme is based on a hyper-chaotic system and two Chebyshev maps. We will use a four dimensional hyper-chaotic system with five system parameters and four initial conditions [29], which can be modeled by Equation (3):

(3) $$\left\{ \begin{array}{l} dx/dt=a(y-x)+w\\ dy/dt=dx-xz+cy\\ dz/dt=xy-bz\\ dw/dt=yz+ew \end{array}\right.$$

where, *a*, *b*, *c*, *d* and *e* are parameters of the system. When *a* = 35, *b* = 3, *c* = 12, *d* = 7 and *e* ∈ (0.085, 0.798), the system is hyper-chaotic and has two positive Lyapunov exponents, *LE*1 = 0.596, *LE*2 = 0.154. So the system is in a hyper-chaotic state. The system attractor curves are presented in Figure 2.

The two Chebyshev maps are modeled by Equation (4):

(4) $$\{\begin{array}{c} u_{1}\left(i+1\right)=\cos\left(4\times \arccos\left(u_{1}\left(i\right)\right)\right)\\ u_{2}\left(i+1\right)=\cos\left(4\times \arccos\left(u_{2}\left(i\right)\right)\right) \end{array}$$

where, *u*_1_(1) and *u*_2_(1) are initial values.

### 2.3. The Generation of Random Sequences of the Encryption System

**Figure 3:**
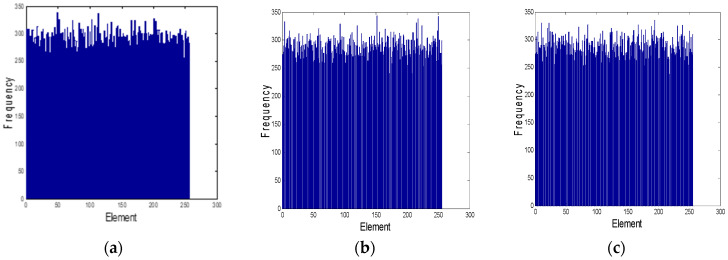
Histogram of three CPRNG sequences. (**a**) The histogram of sequence *S*; (**b**)The histogram of sequence *V*; (**c**)The histogram of sequence T.

The initial values of the chaotic system are given, then we iterate the hyper-chaotic system (1) to produce four sequences denoted as *X* *=* [*x*(*i*)], *Y=* [*y*(*i*)], *Z =* [*z*(*i*)] and *W* *=* [*w*(*i*)], respectively, where, *i* = 1, 2, … At the same time, *u*_1_(1) and *u*_2_(1) are given, the two Chebyshev maps from Equation (4) are iterated to generate two sequences denoted as *U*_1_ and *U*_2_, respectively. To further enhance the complexity of sequences, These six chaotic sequences *X*, *Y*, *Z*, *W*, *U*_1_ and *U*_2_ are transformed into three real value sequences *D*_1_, *D*_2_ and *D*_3_ in the interval [0, 1] by the following Formulas (5)–(7), then transform three real value sequences *D*_1_, *D*_2_ and *D*_3_ into three integer value sequences *S, V* and *T* by the following Formulas (8)–(10), so we get three sequences *S* = {*s*(1), *s*(2), …, *s*(*l*)}, *V =* {*v*(1), *v*(2), …, *v*(*l*)}, *T =* {*t*(1), *t*(2), …, *t*(*l*)}, which will be used in the later encryption process, where, *s*(*i*)*, v*(*i*) and *t*(*i*) ∈ {0, 1, …, 255}, *i* = 1,2, …, *l*:

(5) $$D_{1}=\cos^{2}\left((X+Y+Z)/3\right)$$

(6) $$D_{2}=\cos^{2}\left((W+U_{1}+U_{2})/3\right)$$

(7) $$D_{3}=\cos^{2}\left((X+Z+U_{2})/3\right)$$

(8) $$S=\mathrm{mod}\left(\mathrm{round}(D_{1}\times 10^{15}),256\right)$$

(9) $$V=\mathrm{mod}\left(\mathrm{round}(D_{2}\times 10^{15}),256\right)$$

(10) $$T=\mathrm{mod}\left(\mathrm{round}(D_{3}\times 10^{15}),256\right)$$

where, round(*x*) rounds *x* to the nearest integer, and mod(*x*, *y*) returns the remainder after *x* is divided by *y*. The sequence *D*_1_ is used to scramble images, while *D*_2_, *S*, *V* and *T* are used for image diffusion operation. Figure 3 is the numerical distribution curve of chaotic key sequence *S*, *V* and *T*. the abscissa represents 256 gray levels and the ordinate represents the frequency of each gray level. From Figure 3, it can be seen that the key flow *S*, *V* and *T* distribute evenly, and the pseudo-randomness is good.

### 2.4. Statistical Test Analysis of the Three CPRNG Sequences S, V and T

In order to measure randomness of the three CPRNG sequences *S*, *V* and *T*, we use the NIST SP800–22 statistical test suite (Rev1a, Information Technology Laboratory, Computer Security Resource Center, Gaithersburg, MD, USA), which consists of 15 statistical tests. Each test result is converted to a *p*-value for judgement, and when applying the NIST test suite, a significance level *α* = 0.01 is chosen for testing. If the *p*-value ≥ *α*, then the test sequence is considered to be pseudo-random.

Setting different initial conditions of chaotic system, and using systems (3) and (4) as well as Equations (5)–(10), 1000 sequences *S*, 1000 sequences *V* and 1000 sequences *T* are generated, respectively. The parameters used in the test are set as: *a* = 35, *b* = 3, *c* = 12, *d* = 7, *e* = 0.1583, *x*(0) = 0.398, *y*(0) = 0.45, *z*(0) = 0.78, *w*(0) = 0.98, *u*_1_(1) = 0.58 and *u*2(1) varies from 0.0005 to 0.9995 with a variable step size of 0.0001. Hence, 1000 sequences of {*S*, *V*, *T*} can be generated. The length of each integer sequence is 125,000 and each integer has 8 bits. Then three decimal integer sequences are turned into three binary sequences by converting each decimal number into an 8-bit binary number and connecting them together. Therefore, each binary sequence has the length of 1,000,000 bits (125,000 × 8 = 100,000). Unlike the bit sequence generation method introduced in the related literature [30], the method of generating bit sequences in our scheme can be demonstrated by the following simple example. Suppose the decimal integer sequence *S* has three 8-bit integers, *S* = [23, 106, 149], where, 23 = (0001 0111)_2_, 106 = (0110 1010)_2_, 149 = (1001 0101)_2_. Then the binary sequence *S*’ corresponding to the decimal integer sequences *S* has the following form: *S*’ = [0 0 0 1 0 1 1 1 0 1 1 0 1 0 1 0 1 0 0 1 0 1 0 1]. 15 statistical items (some items include two sub indicators) were tested by using the NIST SP800-22 suite, and the results from all statistical tests are given in Table 1. From Table 1, we can see that all the *p*-values from all 1000 sequences are greater than the significance level *α* = 0.01, indicating that the tests meet the requirements of SP800-22 randomness, and the pass rate is also in acceptable range. Compared with the results of relevant literature [30], the overall result is not very different. However, the linear complexity index of our scheme is obviously better than that of reference [30], but the Rank index is slightly worse than that of reference [30].

## 3. Architecture of the Proposed Cryptosystem

In this paper, we use the classical permutation-diffusion image encryption structure. During the permutation process, we use the permutation sequence generated by the chaotic system to shuffle the pixels. However, the permutation does not change the pixel value, but makes the statistical relationship between cyphertext and key complicated, so that the opponent cannot infer the key statistics from the statistical relationship between cyphertext. Diffusion means that each bit of the plaintext affects many bits of the cyphertext, or that each bit of the cyphertext is affected by many bits of the plaintext, thus enhancing the sensitivity of the cyphertext.

### 3.1. Encryption Algorithm

**Figure 4:**
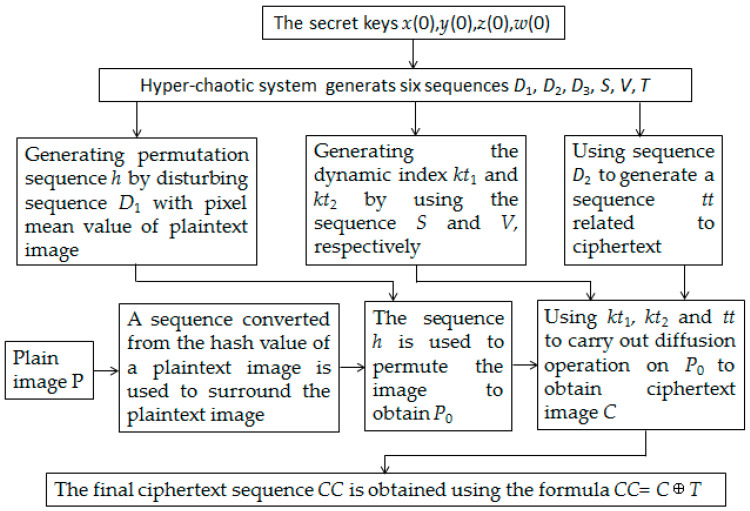
The architecture of the proposed encryption algorithm.

The encryption process consists of three stages. Firstly, generating key streams by using the hyper-chaotic system and adding surrounding pixels to the plaintext image. Secondly, performing the permutation process. Thirdly, performing the diffusion process. The architecture of the encryption process is shown in Figure 4, and the operation procedures are described as follows:

*Step 1*: Assume that the size of the plaintext image is *m* × *n*, adding surrounding pixels to the plaintext image matrix *P_m_*_×*n*_ According to the method described in Section 2.1 to get image matrix *P*′_(*m*+2)×(*n*+2)_. The matrix *P*′_(*m*+2)×(*n*+2)_ is converted to a one dimensional vector *P*_0_ = {*p*_0_(1), *p*_0_(2),…, *p*_0_(*l*)}, where *l* = (*m* + 2) × (*n* + 2).

*Step 2*: Produce the required chaotic sequences *D*_1_, *D*_2_, *S*, *V* and *T* of length *l* for encryption according to the method described in Section 2.3.

*Step 3*: Permuting *P*_0_ obtained in step 1 according to Equations (11) and (12). In order to make the scrambling sequence related to plaintext to prevent the chosen plaintext attack, a disturbance term *g* associated with the plaintext is added according to Equation (10) when the scrambling sequence *h* is generated, where *g* = *sum*(*P*_0_)/(256 × *l*), Therefore, the scrambling sequence *h* = {*h*(1), *h*(2), …, *h*(*l*)} is different when encrypting different plaintext images. In Equation (11), *floor*(*x*) rounds *x* to the nearest integers towards minus infinity:

(11) $$h(i)=i+\mathrm{mod}[floor(D_{1}(i)\times g\times 10^{14}),l-i],i=1,2,3,\ldots ,l.$$

(12) $$\left\{ \begin{array}{l} temp=p_{0}(i)\\ p_{0}(i)=p_{0}(h(i))\\ p_{0}(h(i))=temp \end{array}\right.,i=1,2,3,\ldots ,l.$$

*Step 4*: Perform confusion and diffusion. Encrypt the first element in *p*_0_ by Equation (13):

(13) $$c(1)=\mathrm{mod}((p_{0}(1)+s(1),256)⊕\mathrm{mod}((t(1)+v(1)),256).$$

*Step 5*: Set *i* = 2, 3, …, *l*, calculate the dynamic indexes *kt*_1_ and *kt*_2_ by Equations (14) and (15), which are used for encrypting the *i*-th element in *p*_0_. Obviously, *kt*_1_(*i*) ∈ [1, *i* − 1], *kt*_2_(*i*) ∈ [*i* + 1, *l*]:

(14) $$kt_{1}(i)=floor(s(i)/256\times (i-1))+1,$$

(15) $$kt_{2}(i)=floor(v(i)/256\times (l-i-1))+i+1.$$

*Step 6*: Encrypt the *i*-th element according to the following Equations (16)–(18):

(16) $$tt(i)=\mathrm{mod}(floor(D_{2}(i)\times c(i-1))\times 10^{4},256),i=1,2,3,\;\ldots ,l-\;1.$$

(17) $$c(i)=\mathrm{mod}((p_{0}(i)+c(kt_{1}(i))),256)⊕\mathrm{mod}((tt(i)+p_{0}(kt_{2}(i))),256),i=1,2,\ldots ,l-\;1.$$

(18) $$c(l)=\mathrm{mod}((p_{0}(l)+c(kt_{1}(l))),256)⊕tt(l)$$

From Equation (16), for different plain images, the sequence [*tt*(*i*)] will be different, that will lead to the different *i*-th encrypted value.

*Step7*: The final cyphertext sequence *CC* = [*cc*(1), *cc*(2), …, *cc*(*l*)] is obtained by using Equation (19). Transform the diffused vector *CC* into the *m* × *n* matrix, then the cypher image is obtained:

(19) cc(i)=c(i)⊕t(i)

### 3.2. Decryption Algorithm

The decryption process is the process of transforming cyphertext into plaintext, and the reverse process of encryption. The decryption process is described as follows:

*Step 1*: Produce the required chaotic sequences *D*_1_, *D*_2_, *S*, *V* and *T* of length *l* for decryption according to the method described in Section 2.3 and calculate the dynamic indexes *kt*_1_ and *kt*_2_ according to Equations (14) and (15).

*Step 2*: The cyphertext image is translated into a one dimensional vector *CC* = [*cc*(1), *cc*(2), …, *cc*(*l*)]. The intermediate cyphertext C is obtained by:

(20) c(i)=cc(i)⊕t(i)

*Step 3*: Calculate the sequence *tt* according to Equation (16) and decrypt the last element in *p*_0_ by:

(21) $$p0(l)=\mathrm{mod}(c(l)⊕tt(l)-c(kt_{1}(l)),256)$$

*Step 4*: In the opposite direction, we decrypt the plaintext pixel *P*_0_(*l* − 1), *P*_0_(*l* − 2), …, *P*_0_(2) by Equation (22). Finally, the pixel *P*_0_ (1) is decrypted as:

(22) $$p_{0}(i)=\mathrm{mod}(c(i)⊕\mathrm{mod}(tt(i)+p_{0}(kt_{2}(i)),256)-c(kt_{1}(i)),256),i=l-\;1,l-\;2,l-\;3,\;\ldots ,2.$$

(23) $$p_{0}(1)=\mathrm{mod}(c(1)⊕\mathrm{mod}(t(1)+v(1)),256)-s(1),256)$$

*Step 5*: Perform inverse permutation. Because the sum of pixel values before and after scrambling remains unchanged, the *g* value can be calculated by the sequence *P*_0_ decrypted in Step 4, Thus, the sequence *H* = {*h*(1), *h*(2), …, *h*(*l*)} can be obtained by Equation (11). It should be noted that this process is reversed in the direction of encryption, from the last pixel to the first pixel, that is:

(24) $$\left\{ \begin{array}{l} temp=p_{0}(i)\\ p_{0}(i)=p_{0}(h(i))\\ p_{0}(h(i))=temp \end{array}\right.,i=l-\;1,l-\;2,l-\;3,\;\ldots ,\;1.$$

Finally, the decrypted sequence *P*_0_ is transformed into a matrix *P*′ of size (*m* + 2) × (*n* + 2). Discarding the first row, the last row, and the first column and the last column of the matrix *P*′, and we can obtain a matrix *P* of size *m* × *n. P* is the recovered plaintext image.

### 3.3. Application of the Algorithm for Color Images

A color image is composed of three main components, i.e., *R*, *G* and *B*. The hash values of *R*, *G* and *B* matrices are computed respectively, and then the hash values are transformed into sequences according to the method of Section 2.1. Then adding surrounding pixels to *R*, *G* and *B* by using the sequences to obtain three new matrices *R*′, *G*′ and *B*′, respectively. Then *R*′, *G*′ and *B*′ are encrypted in parallel and similar to the encryption of gray level image. Decryption process of matrixes *R*, *G* and *B* is also similar to the proposed decryption process in Section 3.2.

### 3.4. The Advantages in the New Encryption Scheme

(1) The method of surrounding pixels generated by the SHA-256 hash value of the plaintext image is adopted, which can enhance the ability of the encryption system to resist chosen plaintext attacks. In general, selecting an image of all the same pixel values to chosen plaintext attack, which can eliminate the global scrambling effect. But in the new encryption algorithms, even encrypt an image of all the same pixel values, because the first step is to add surrounding pixels to the image, then the image is not an image of all the same pixel values. On the other hand, the hash value of the image is not needed in decryption, which reduces the difficulty of key management.
(2) In the permutation process, by adding a perturbation *g* (*g* = sum(*P*_0_)/(256 × *l*)) to the chaotic sequence *D*_1_, the permutation sequence *h* is generated by Equation (10). Therefore, *h* is related to plaintext, which can resist the chosen plaintext attack. At the same time, *g* is not part of the decryption key, which reduces the difficulty of key management.
(3) From Equation (16), it is known that the sequence *tt* is related to the transition cyphertext *c*, so the sequence *tt* is different when encrypting different images, which further strengthens the ability of the encryption system to resist chosen plaintext attack.
(4) From the cyphertext feedback mechanism of Equation (17), It can be seen that our encryption algorithm is sensitive to plaintext.

## 4. Simulation Results

**Figure 5:**
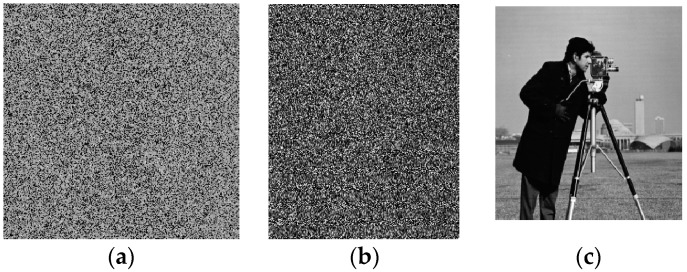
Experimental results. (**a**) scrambled image; (**b**) cyphertext image; (**c**) decrypted plaintext image.

In this paper, the standard 256 × 256 image of “cameraman” is used as the input image. Matlab 2014a (MathWorks, Natick, MA, USA) is utilized to simulate the encryption and decryption operations and set parameters (*x*(0), *y*(0), *z*(0), *w*(0)) = (0.398, 0.456, 0.784, 0.982). The continuous hyper-chaotic system (3) was solved by the ode45 solver of Matlab. The time step used in this algorithm is the adaptive variable step size instead of fixed step size. Figure 5a is the scrambled image, Figure 5b is the encrypted image and the decrypted image is shown in Figure 5c. It can be seen that the cyphertext image is a chaotic image, and has nothing to do with the original image. Therefore, the encryption effect of the algorithm is good.

## 5. Security Analysis

In this section, we will discuss the security analysis of the proposed encryption scheme with the traditional 8-bit gray image as an example.

### 5.1. Key Space

In cryptography, the larger the key space, the stronger the ability to resist brute force attacks. In the proposed cryptosystem, the keys are the initial value *x*(0), *y*(0), *z*(0), *w*(0) of the chaotic system (3), the chaotic system parameter *e* and the initial values *u*_1_(1), *u*_2_(1) of the two Chebyshev maps (4). The precision of *x*(0), *y*(0), *z*(0), *w*(0), *u*_1_(1) and *u*_2_(1) is 10^−15^, while the precision of the parameter *e* is 10^−^^12^ for *e*∈(0.085, 0.798), so the key space size will be (10^15^)^6^ × 10^12^ = 10^102^ ≈ 2^339^. In Reference [1], Li pointed out that the effective key space of the image encryption system should be greater than 2^100^ in order to prevent brute force attacks, so the key space of our algorithm is sufficiently large to resist against brute-force attacks.

Table 2 lists the key space of several similar algorithms. By comparison, the key space of this algorithm is better than most algorithms’ key space. The size of the key space depends not only on the number of keys, but also on the number of possible values for each key. The problem of numerical chaotic systems is that the finite precision of the machines (e.g., computers) leads to performance degradation [31,32,33,34], such as: the key space is reduced, some weak keys appear, and the randomness of the sequence is reduced. In order to identify and avoid weak keys, we need to calculate the Lyapunov exponents of chaotic systems, or plot the phase space trajectories of the system.

### 5.2. Key Sensitivity

The key sensitivity can be evaluated in two aspects: First, the cyphertext image will be completely different when encrypting the same plaintext image with slightly different keys, which is measured by the change rate *t* of the cyphertext image. Second, no information about the plaintext image is available in the image decrypted from the wrong key, even though there is a very small difference between the wrong key and the correct key.

The specific method of calculating the change rate t of cyphertext image is as follows: first, the change of the key is *kh*, and the other parameters remain unchanged. For example, when calculating the sensitivity of the key *a*, the cyphertext *C*_1_ is obtained by encrypting the plaintext image with the key *a*. In the same way, the cyphertext image *C*_2_ and the cyphertext image *C*_3_ are obtained by encrypting the plaintext image with the key *a* + *kh* and the key *a* − *kh*, respectively. We can calculate the pixel difference rate *t*_1_ between *C*_1_ and *C*_2_ and the pixel difference rate *t*_2_ between *C*_1_ and *C*_3_, respectively. Then, the change rate of cyphertext image *t* = (*t*_1_ + *t*_2_)/2 is obtained. The sensitivity of each key is calculated by this method, as shown in Table 3, where the change *kh* is 10^−15^ for each key *x*(0), *y*(0), *z*(0), *w*(0). The calculated results show that the new algorithm is very sensitive to the initial secret key values.

**Figure 6:**
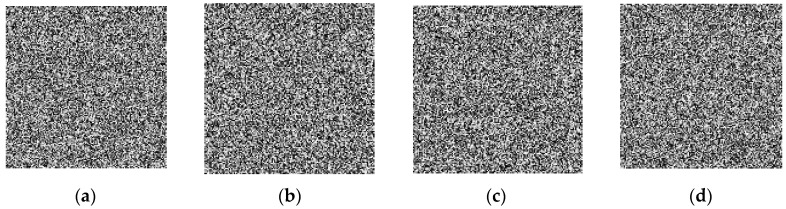
The image decrypted by the wrong keys. (**a**) decryption result of key *Key*1; (**b**) decryption result of *Key*2; (**c**) decryption result of *Key*3; (**d**) decryption result of *Key*4.

The sensitivity test results of Table 3 confirmed that the sensitivity of the proposed algorithm to the keys *x*(0), *y*(0), *z*(0), *w*(0) is very high, and can reach more than 10^−15^. In order to evaluate the sensitivity of the key in the second aspects, we select the following error keys *Key*1, *Key*2, *Key*3 and *Key*4 to decrypt the original cyphertext image, and the decryption result is shown in Figure 6.

*Key*1 = {*x*(0), *y*(0), *z*(0), *w*(0)} = (0.398 + 10^−15^, 0.456, 0.784, 0.982),

*Key*2 = {*x*(0), *y*(0), *z*(0), *w*(0)} = (0.398, 0.456 + 10^−15^, 0.784, 0.982),

*Key*3 = {*x*(0), *y*(0), *z*(0), *w*(0)} = (0.398, 0.456, 0.784 + 10^−15^, 0.982),

*Key*4 = {*x*(0), *y*(0), *z*(0), *w*(0)} = (0.398, 0.456, 0.784, 0.982 + 10^−15^).

### 5.3. Plaintext Sensitivity

The sensitivity of the algorithm to plaintext means that a small change in plaintext will cause a huge change in the corresponding cyphertext. This is one of the criteria for cryptographic security analysis. From the encryption process, we can see that the algorithm is sensitive to plaintext: first, if the image has a little difference, the hash value will be completely different. Therefore, the two matrices *RI* and *CI* generated in Section 2.1 and the disturbance term *g* will be different, so the scrambled image will be different and will lead to the great change of pseudo-random sequence *tt*. Experimentally, *NPCR* (number of pixels change rate) and *UACI* (unified average changing intensity) are used to measure the degree of sensitivity of image encryption algorithms to plaintext. *NPCR* and *UACI* respectively represent the percentage and change degree of the number of pixels in the encrypted image after the pseudo-random change of the gray value of a pixel in the original image. The formulas for the calculation of *NPCR* and *UACI* are as follows:

(25) $$NPCR=\frac{1}{M\times N}\sum_{i=1}^{M}\sum_{j=1}^{N}D(i,j)\times 100\%,$$

(26) $$UACI=\left(\sum_{i=1}^{M}\sum_{j=1}^{N}\left(\frac{\left|x(i,j)-x’(i,j)\right|}{255\times M\times N}\right)\right)\times 100\%,$$

(27) $$D(i,j)=\{\begin{array}{c} 1,\;if\;x(i,j)\neq x’(i,j)\\ 0,\;if\;x(i,j)=x’(i,j) \end{array}.$$

where, *M* × *N* is the size of the image. *x*(*i*, *j*) represent the pixel in a coordinate (*i*, *j*) of the cyphertext image corresponding to the original plaintext image, and *x*′(*i*, *j*) represent the pixel in a coordinate (*i*, *j*) of the cyphertext image corresponding to the changed plaintext image. For 256 bit grayscale images, the expected values of *NPCR* and *UACI* are 99.6094% and 33.4635%, respectively. In this paper, four classical images (“cameraman”, “pepper”, “rice” and “autumn”) are selected to be tested. In each image, the pixel values of randomly selected 200 pixels are changed, and their maximum, minimum, and average values of *NPCR* are listed in the Table 4. In order to show the superiority of our encryption algorithm, the maximum and minimum values of *NPCR* and *UACI* for each image calculated by the encryption algorithm of Referemce [35] are shown in Table 5.

As shown in Table 4 and Table 5, the average values of *NPCR* and *UACI* of the four images of the new algorithm are higher than the average of *NPCR* and *UACI* in Reference [31], thus proving that our algorithm has better performance in resisting differential attacks.

### 5.4. Statistical Analysis

Statistical analysis mainly includes: histogram analysis, chi-square test, adjacent pixel correlation analysis and information entropy analysis. In this part we will evaluate the algorithm from above aspects.

#### 5.4.1. Statistical Histogram Analysis

**Figure 7:**
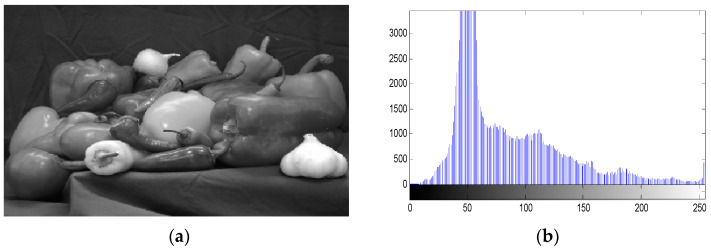

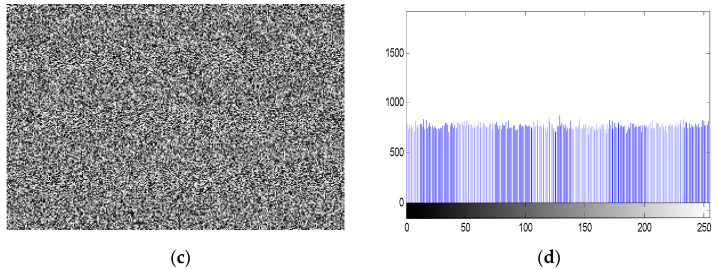
Histogram analysis. (**a**) The plaintext image of “pepper”; (**b**) the histogram of the plaintext image; (**c**) the cyphertext image; (**d**) the histogram of the cyphertext image.

Gray histogram is a function of gray level, which reflects the distribution of gray level in the image and describes the number of pixels of each gray level in the image, but does not contain the position information of these pixels in the image. Figure 7 are the histograms of the Pepper image and the corresponding cipher images. In the histogram, the horizontal axis denotes the gray level, and the vertical axis denotes the pixel number of each gray level. It can be seen that the probability distribution of plaintext image histogram presents a single peak distribution, while the corresponding cyphertext image histogram probability distribution is close to the equal probability distribution, so the cyphertext image is a pseudo-random image.

#### 5.4.2. Chi-Square Test

Figure 7d shows that the histogram of the cyphertext image is uniformly distributed, which can resist statistical attacks and can be proved by the chi-square test [36], which is described by the following expression:

(28) $$x^{2}=\sum_{k=1}^{256}\frac{{\left(v_{k}-e\right)}^{2}}{e}$$

where, *v_k_* is the actual frequency of each gray level, and e is the expected frequency of each gray level. For different sizes of images, *e* is different, for example, *e* is 256 for the image cameraman of size 256 × 256. however, *e* is 768 for the image pepper of size 384 × 512. The smaller the chi square value, the better the uniformity of cyphertext images. For the confidence level a = 0.05, if the chi square value does not exceed 295.25, it is considered to pass the test. In this paper, the cyphertext image is generated by changing one bit of the ordinary image. The process is repeated 30 times. for confidence level = 0.05, and the average results for the four images “cameraman”, “pepper”, “rice” and “autumn” are demonstrated in Table 6.

It can be seen that the chi-square value of the histogram of the cyphertext images are less than 295.5, which means that the histogram of the cyphertext image has passed Chi-square test for confidence level =0.05 and better than in Kulsoom [37].

#### 5.4.3. Information Entropy

The entropy of an image is expressed as the average number of bits of the set of gray levels of an image. It also describes the average amount of information of an image source. The greater the entropy, the more confusing the information provided by the image. For discrete two-dimensional images, the formula of information entropy *E* is shown in Equation (29):

(29) $$E=-\sum_{i=0}^{n}p_{i}\log_{2}(p_{i})$$

where, *p_i_* is the probability of the occurrence of gray value *i*. When the probability distribution of cyphertext is equal probability distribution, that is, the probability of each value of [0, 255] is 1/256, the maximum entropy is 8 bits. The information entropy of four cyphertext images of “rice”, “cameraman”, “autumn” and “pepper” is listed in the second column of Table 7, At the same time, the information entropy of the cyphertext image obtained by the other algorithms in [38,39,40,41,42] are listed in columns 3–7 of Table 7. It can be seen that the information entropy of the encrypted image of the four images is very close to 8 bits and our algorithm has greater superiority.

#### 5.4.4. Pixel Correlation Analysis

**Figure 8:**
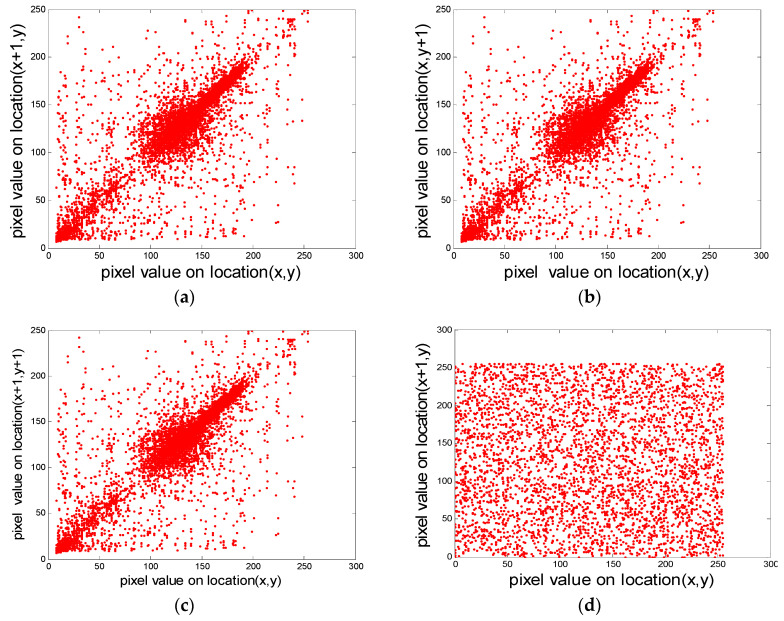

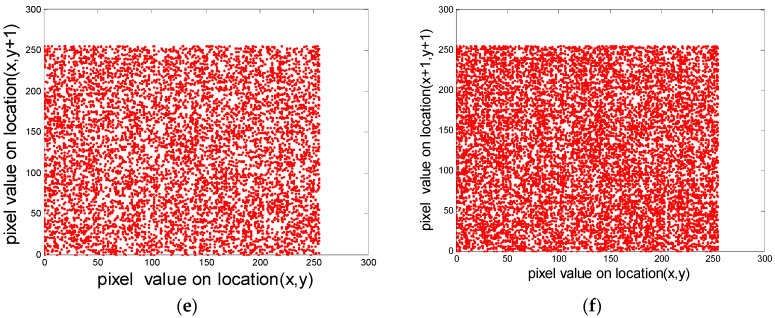
The correlation plots of the cameraman image and the corresponding ciphered image of cameraman. (**a**) Horizontal correlation of the cameraman image; (**b**) Vertical correlation of the cameraman image; (**c**) Diagonal correlation of the cameraman image; (**d**) Horizontal correlation of the cyphered image of cameraman; (**e**) Vertical correlation of the cyphered image of cameraman; (**f**) diagonal correlation of the cyphered image of cameraman.

In a natural image, there is a high correlation between each pixel and its adjacent pixels, which means that there is a small difference in the gray value in the larger area of the image. One of the goals of encrypted image is to reduce the correlation between adjacent pixels, and the smaller the correlation, the better the encryption effect, the higher the security. Correlation mainly includes the correlation between horizontal pixels, vertical pixels and diagonal pixels. Firstly, 4000 pixels are selected randomly from the “cameraman” image and the corresponding cyphertext image as the base points, and 4000 pairs of adjacent pixels are collected along the horizontal, vertical and diagonal directions respectively, and the correlation distribution maps in these three directions are drawn, as shown in Figure 8. It can be seen that there is a strong correlation between adjacent pixels of plaintext image, showing a linear relationship, and for cyphertext image, this correlation is greatly weakened, showing a strong randomness. This indicates that the image encryption effect is good and the security is high.

In order to further quantify the linear correlation between the adjacent pixels, the correlation coefficients can be calculated as:

(30) $$xc=\frac{n\sum_{i=1}^{n}x_{i}y_{i}-\sum_{i=1}^{n}x_{i}\sum_{i=1}^{n}y_{i}}{\sqrt{n\sum_{i=1}^{n}x_{i}{}^{2}-{\left(\sum_{i=1}^{n}x_{i}\right)}^{2}}\sqrt{n\sum_{i=1}^{n}y_{i}{}^{2}-{\left(\sum_{i=1}^{n}y_{i}\right)}^{2}}}$$

where, *x_i_* and *y_i_* represent the gray values of two adjacent pixels, respectively, and *n* represents the number of pixel pairs selected. The correlation coefficients of adjacent pixels of the original image and the cyphertext image are shown in Table 8. It can be seen that the absolute values of the correlation coefficients between adjacent pixels in three directions of the plaintext image are very close to 1, while the absolute values of the correlation coefficients between adjacent pixels in each direction of the corresponding cyphertext image are close to 0, and the correlation is weakened.

### 5.5. Computational Speed Analysis

Finally, the time complexity of the algorithm for encryption/decryption is evaluated. Several images for different sizes have been considered and the time complexity is given. The time complexity analysis is achieved on zn Intel(R) Pentium(R) Dual Core processor CPU (2.3 GHz with 2 GB RAM) personal computer. The algorithm is developed in Matlab R2014a and compiled by 7.14 on Windows 7 Home Premium edition. The results are shown in Table 9. This shows that our algorithm is faster than most of the algorithms in [39,40,43,44], but it is just slightly slower than that in [40]. In general, the encryption algorithm based on the spatial domain is faster than the algorithm based on the image frequency domain [45]. 

## 6. Conclusions

A new image encryption scheme is proposed, which includes three main components: adding pixels around the image, pixel scrambling and pixel diffusion. Firstly, the hash value of the plaintext image is converted into a pseudo-random sequence, then adding pseudo-random sequences to the surrounding area of plaintext images. The pseudo-random sequences used in the permutation and diffusion process are related to the plaintext image, which can resist chosen plaintext attack. Different from previous algorithms, our algorithm transforms the hash value of the plaintext image into a pseudo-random sequence, which takes the pseudo-random sequence as part of the encrypted image, while the previous algorithm takes the hash value of the plaintext image as a part of the key. In our encryption algorithm, the key of the encryption system is only the initial value of the chaotic system, which reduces the difficulty of key management. In addition, the algorithm also has the following advantages, which can be demonstrated by theoretical analysis and experimental results: the key space is large, the cyphertext is very sensitive to plaintext and keys, the distribution of pixels in encrypted image is uniform, the correlation between adjacent pixels of cyphertext is very low, and the information entropy of cyphertext images is close to the ideal value of 8, Therefore, the proposed algorithm has good application prospects in secure image communication and storage applications.

## Figures and Tables

**Table 1:** NIST SP800-22 standard test of pseudo-random sequence *S*′, *V*′ and *T*′.

Statistical Test Name	*S*′		*V*′		*T*′	
	Pass Rate	*p*-Value	Pass Rate	*p*-Value	Pass Rate	*p*-Value
Frequency(monobit)	99.5%	0.9346	99.3%	0.4058	99.4%	0.4708
Block Frequency	99.2%	0.8068	99.1%	0.6079	99.0%	0.5485
The Run Test	99.5%	0.4088	99.6%	0.4317	99.5%	0.5493
Longest Run of Ones	98.6%	0.1481	98.8%	0.4555	98.6%	0.4419
Rank	98.5%	0.0465	98.3%	0.0467	98.1%	0.0103
DFT Spectral	99.3%	0.9537	99.1%	0.5365	99.3%	0.6539
Non-Overlapping Templates	99.1%	0.6163	99.0%	0.5348	98.8%	0.4807
Overlapping Templates	98.8%	0.7597	98.6%	0.5331	98.4%	0.6420
Universal Statistical Test	98.5%	0.5825	98.3%	0.4624	98.2%	0.4171
Linear Complexity	98.9%	0.2215	98.7%	0.4642	98.5%	0.4936
Serial Test 1	99.1%	0.3358	98.9%	0.2421	98.7%	0.2602
Serial Test 2	99.2%	0.2046	99.4%	0.4207	99.3%	0.2315
Approximate Entropy	98.8%	0.7522	98.6%	0.6033	98.8%	0.4784
Cumulative Sums (forward)	99.6%	0.4752	99.8%	0.8023	99.7%	0.8163
Cumulative Sums (Reverse)	99.4%	0.8898	99.2%	0.6596	99.3%	0.8101
Random Excursions	98.7%	0.1599	98.8%	0.1713	98.6%	0.1314
Random Excursions Variant	98.9%	0.3226	98.4%	0.1564	98.6%	0.0942

**Table 2:** Key space comparisons.

Encryption Algorithm	Key Space
Proposed scheme	2^339^
Reference [24]	2^149^
Reference [25]	2^256^
Reference [31]	2^299^
Reference [32]	2^375^
Reference [33]	>2^128^
Reference [34]	2^357^

**Table 3:** Sensitivity tests for each initial secret key value.

Keys	Change Rate of Cyphertext Image *t*
*x*(0)	0.9963
*y*(0)	0.9964
*z*(0)	0.9976
*w*(0)	0.9975

**Table 4:** *NPCR* and *UACI* test results of slight change of plaintext in our algorithm.

Images		Rice	Autumn	Pepper	Cameraman
*NPCR*%	Max	99.8943	99.7932	99.9012	99.7821
	Min	99.5426	99.4213	99.3809	99.4608
	Average	99.6062	99.6115	99.5956	99.5697
*UACI*%	Max	33.5698	33.7754	33.8712	33.6590
	Min	33.3216	33.5500	33.4919	33.1958
	Average	33.4419	33.6319	33.5418	33.3618

**Table 5:** *NPCR* and *UACI* test results of slight change of plaintext in [35].

Images		Rice	Autumn	Pepper	Cameraman
*NPCR*%	Max	99.8812	99.6623	99.8719	99.8864
	Min	99.4961	99.5512	99.5698	99.5091
	Average	99.6006	99.6098	99.5796	99.5692
*UACI*%	Max	33.5612	33.6067	33.8523	33.7019
	Min	33.3187	33.5602	33.4967	33.2195
	Average	33.4297	33.5897	33.5154	33.3478

**Table 6:** Chi-test results of 30 encrypted images under confidence level is 0.05.

Test Images	*x*^2^ of Plain Image	*x*^2^ of Cypherimage in [33]	*x*^2^ of Cypherimage in Our Algorithm
cameraman	16,711,680	288.9823 < 295.25	285.3125 < 295.25
pepper	50,135,040	269.3387 < 295.25	260.3421 < 295.25
rice	96,312	284.2387 < 295.25	278.6172 < 295.25
autumn	18,122,850	289.9832 < 295.25	288.5792 < 295.25

**Table 7:** Entropy of cyphertext images.

Images	This Paper	Ref. [34]	Ref. [39]	Ref. [40]	Ref. [41]	Ref. [42]
Rice (256 × 256)	7.9973	7.9864	7.9936	7.9643	7.9875	7.9968
cameraman (256 × 256)	7.9989	7.9763	7.9952	7.9867	7.9946	7.9865
autumn (206 × 345)	7.9968	7.9564	7.9962	7.9698	7.9864	7.9972
pepper (512 × 512)	7.9992	7.9819	7.9983	7.9949	7.9896	7.9993

**Table 8:** Correlation coefficients between adjacent elements of original image and encrypted image.

Images	Horizontal	Vertical	Diagonal
Plaintext “Rice”	0.9427	0.9263	0.8994
Cyphertext “Rice”	−0.0046	0.0287	−0.0361
Plaintext “Cameraman”	0.9588	0.9360	0.9095
Cypher “Cameraman”	−0.0017	−0.0279	0.0047
Plaintext “autumn”	0.9675	0.9845	0.9821
Cyphertext “autumn”	−0.0087	0.0142	0.0098
Plaintext “pepper”	0.9894	0.9931	0.9847
Cyphertext “pepper”	−0.0055	−0.0194	−0.0295

**Table 9:** Comparison of encryption or dcryption time (EDT) of 8-bit gray level images for different image sizes.

Image Size (*M* × *N*)	Ref. [39]	Ref. [40]	Ref. [43]	Ref. [44]	Proposed System
64 × 64	0.07	0.03	0.19	0.61	0.02
128 × 128	0.19	0.08	0.29	2.17	0.06
256 × 256	0.46	0.18	6.01	7.73	0.22
512× 512	1.88	0.97	35.59	31.59	0.85
1024 × 1024	3.62	2.94	253.88	169.21	3.11

## References

[1] Alvarez G., Li S. (2006). Some basic cryptographic requirements for chaos-based cryptosystems. Int. J. Bifurc. Chaos.

[2] Fridrich J. (1998). Symmetric ciphers based on two-dimensional chaotic maps. Int. J. Bifurc. Chaos.

[3] Hsiao H.-I., Lee J. (2015). Color image encryption using chaotic nonlinear adaptive filter. Signal Process..

[4] Mirzaei O., Yaghoobi M., Irani H. (2012). A new image encryption method: Parallel sub-image encryption with hyper chaos. Nonlinear Dyn..

[5] Wu Y., Zhou Y., Agaian S., Noonan J.P. (2014). A symmetric image cipher using wave perturbations. Signal Process..

[6] Patidar V., Pareek N.K., Purohit G., Sud K.K. (2010). Modified substitution-diffusion image cipher using chaotic standard and logistic maps. Commun. Nonlinear Sci. Numer. Simul..

[7] Zhu C. (2012). A novel image encryption scheme based on improved hyperchaotic sequences. Opt. Commun..

[8] Zhang Y., Xiao D., Shu Y., Li J. (2013). A novel image encryption scheme based on a linear hyperbolic chaotic system of partial differential equations. Signal Process. Image Commun..

[9] Zhang W., Wong K.-W., Yu H., Zhu Z.-L. (2013). A symmetric color image encryption algorithm using the intrinsic features of bit distributions. Commun. Nonlinear Sci. Numer. Simul..

[10] Zhang Y., Xiao D. (2014). An image encryption scheme based on rotation matrix bit-level permutation and block diffusion. Commun. Nonlinear Sci. Numer. Simul..

[11] Chai X. (2017). An image encryption algorithm based on bit level brownian motion and new chaotic systems. Multimed. Tools Appl..

[12] Zhu C.-X., Sun K.-H. (2012). Cryptanalysis and improvement of a class of hyperchaos based image encryption algorithms. Acta Phys. Sin..

[13] Ozkaynak F., Yavuz S. (2014). Analysis and improvement of a novel image fusion encryption algorithm based on DNA sequence operation and hyper-chaotic system. Nonlinear Dyn..

[14] Zhu C., Xu S., Hu Y., Sun K. (2015). Breaking a novel image encryption scheme based on brownian motion and pwlcm chaotic system. Nonlinear Dyn..

[15] Yap W.-S., Phan R.C.W., Yau W.-C., Heng S.-H. (2015). Cryptanalysis of a new image alternate encryption algorithm based on chaotic map. Nonlinear Dyn..

[16] Chen L., Ma B., Zhao X., Wang S. (2016). Differential cryptanalysis of a novel image encryption algorithm based on chaos and line map. Nonlinear Dyn..

[17] Li C., Lin D., Lu J. (2017). Cryptanalyzing an image-scrambling encryption algorithm of pixel bits. IEEE Multimed..

[18] Zhu C., Sun K. (2018). Cryptanalyzing and improving a novel color image encryption algorithm using RT-enhanced chaotic tent maps. IEEE Access.

[19] Norouzi B., Mirzakuchaki S., Seyedzadeh S., Mosavi M.R. (2014). A simple, sensitive and secure image encryption algorithm based on hyper-chaotic system with only one round diffusion process. Multimed. Tools Appl..

[20] Diab H., El-semary A.M. (2018). Secure image cryptosystem with unique key streams via hyper-chaotic system. Signal Process..

[21] Akhavan A., Samsudin A., Akhshani A. (2017). Cryptanalysis of an image encryption algorithm based on DNA encoding. Opt. Laser Technol..

[22] Zhang G., Liu Q. (2011). A novel image encryption method based on total shuffling scheme. Opt. Commun..

[23] Zhu C., Liao C., Deng X. (2013). Breaking and improving an image encryption scheme based on total shuffling scheme. Nonlinear Dyn..

[24] Wang X., Zhu X., Wu X., Zhang Y. (2017). Image encryption algorithm based on multiple mixed hash functions and cyclic shift. Opt. Lasers Eng..

[25] Guesmi R., Farah M.A.B., Kachouri A., Samet M. (2016). A novel chaos-based image encryption using DNA sequence operation and secure hash algorithm sha-2. Nonlinear Dyn..

[26] Liu H., Wang X. (2010). Color image encryption based on one-time keys and robust chaotic maps. Comput. Math. Appl..

[27] Huang L., Cai S., Xiao M., Xiong X. (2018). A simple chaotic map-based image encryption system using both plaintext related permutation and diffusion. Entropy.

[28] Chai X., Chen Y., Broyde L. (2017). A novel chaos-based image encryption algorithm using DNA sequence operations. Opt. Lasers Eng..

[29] Li Y., Liu X., Chen G., Liao X. (2011). A new hyperchaotic lorenz-type system: Generation, analysis, and implementation. Int. J. Circuit Theory Appl..

[30] Stoyanov B.P., Todorov M.D. (2013). Pseudo-random bit generator based on chebyshev map. Application of Mathematics in Technical and Natural Sciences.

[31] Li S., Chen G., Mou X. (2005). On the dynamical degradation of digital piecewise linear chaotic maps. Int. J. Bifurc. Chaos.

[32] Li S., Chen G., Wong K.-W., Mou X., Cai Y. (2004). Baptista-type chaotic cryptosystems: Problems and countermeasures. Phys. Lett. A.

[33] Curiac D.-I., Volosencu C. (2012). Chaotic trajectory design for monitoring an arbitrary number of specified locations using points of interest. Math. Probl. Eng..

[34] Curiac D.I., Iercan D., Dranga O., Dragan F., Banias O. Chaos-Based Cryptography: End of the Road?. Proceedings of the International Conference on Emerging Security Information, System and Technologies.

[35] Wang X.-Y., Gu S.-X., Zhang Y.-Q. (2015). Novel image encryption algorithm based on cycle shift and chaotic system. Opt. Lasers Eng..

[36] Khanzadi H., Eshghi M., Borujeni S.E. (2014). Image encryption using random bit sequence based on chaotic maps. Arab. J. Sci. Eng..

[37] Kulsoom A., Xiao D., Aqeel Ur R., Abbas S.A. (2016). An efficient and noise resistive selective image encryption scheme for gray images based on chaotic maps and DNA complementary rules. Multimed. Tools Appl..

[38] Wang X.-Y., Zhang Y.-Q., Bao X.-M. (2015). A novel chaotic image encryption scheme using DNA sequence operations. Opt. Lasers Eng..

[39] Stoyanov B., Kordov K. (2015). Image encryption using chebyshev map and rotation equation. Entropy.

[40] Stoyanov B., Kordov K. (2014). Novel image encryption scheme based on chebyshev polynomial and duffing map. Sci. World J..

[41] Seyedzade S.M., Mirzakuchaki S., Atani R.E. A novel image encryption algorithm based on hash function. Proceedings of the Iranian Conference on Machine Vision and Image Processing.

[42] Chai X.-L., Gan Z.-H., Yuan K., Lu Y., Chen Y.-R. (2017). An image encryption scheme based on three-dimensional brownian motion and chaotic system. Chin. Phys. B.

[43] Wang X., Zhang J. An image scrambling encryption using chaos-controlled poker shuffle operation. Proceedings of the International Symposium on Biometrics and Security Technologies.

[44] Rehman A.U., Liao X., Kulsoom A., Abbas S.A. (2015). Selective encryption for gray images based on chaos and DNA complementary rules. Multimed. Tools Appl..

[45] Ramadan N., Ahmed H.H., El-khamy S.E., Abd El-Samie F.E. (2017). Permutation-substitution image encryption scheme based on a modified chaotic map in transform domain. J. Central South Univ..

